# Effectiveness of Inhalation of a New Essential Oil Formulation on Asthma Through Network Pharmacology and In Vivo Analysis

**DOI:** 10.1002/fsn3.70763

**Published:** 2025-08-07

**Authors:** Mi Hye Kim, Seong Chul Jin, Woong Mo Yang

**Affiliations:** ^1^ College of Korean Medicine Woosuk University Jeonju Republic of Korea; ^2^ Department of Convergence Korean Medical Science, College of Korean Medicine Kyung Hee University Seoul Republic of Korea; ^3^ Korean Medicine Digital Convergence Center Kyung Hee University Seoul Republic of Korea

**Keywords:** *Abies holophylla* Maxim., *Asarum sieboldii* Miq., asthma, essential oils, *Mentha piperita* L., network pharmacology, Th2 cell

## Abstract

Asthma is one of the chronic inflammatory disorders with shortness of breath, chest tightness, wheezing, and coughing. Considering that essential oils from aromatic plants have been widely used for relieving inflammatory diseases, the protective effects and molecular mechanisms of the inhalation of the essential oil formulation derived from *Mentha piperita* L., *Asarum sieboldii* Miq., and *Abies holophylla* Maxim. named FEO‐03 were investigated on asthma based on the network analysis and confirming in vivo study. The target genes from consisting compounds of each essential oil were compared with the gene set of “Asthma”. Through network pharmacology analysis, the potential effects and target mechanisms of FEO‐03 on asthma are closely associated with the T helper 2 (Th2)‐related cytokine interactions. And FEO‐03 was aerosolized by nebulizer for 5 min 3 times per week over 7 weeks in the ovalbumin and particulate matter‐sensitized BALB/c mice. Nebulized FEO‐03 markedly reduced hyperplasia of respiratory epithelium and goblet cell activation in the trachea and lung. The number of inflammatory cells in BALF and serum immunoglobulin levels were significantly down‐regulated in the FEO‐treated mice. In addition, FEO‐03 regulated expressions of Th2‐related cytokines with the periostin expressions. Furthermore, the fibrotic region of lung tissues was significantly decreased along with the cadherin switch markers by the FEO‐03 treatment. Taken together, FEO‐03, a formulation of essential oils, could ameliorate the fibrosis by inhibiting Th2‐specific cytokines and periostin derived epithelial‐mesenchymal transition in asthma.

## Graphical abstract

Formula essential oil (FEO)‐03, which consists of *Mentha piperita* L., *Asarum sieboldii* Miq., and *Abies holophylla* Maxim. essential oils, is newly developed for relieving asthma. Through network pharmacology analysis and animal experiments, FEO‐03 was found to inhibit the Th2‐related inflammatory and fibrotic responses in airway tissues via regulation of periostin/TGF‐β signaling pathway in asthma.

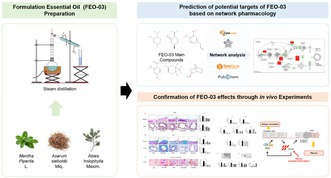

**Abbreviations**
BALF: bronchoalveolar lavage fluid
DEX: dexamethasone
ELISA: enzyme‐linked immunosorbent assay
EMT: epithelial‐to‐mesenchymal transition
FEO‐03: formulation essential oil‐03
H&E: hematoxylin and eosin
OVA: ovalbumin
PAS: periodic acid‐Schiff
PM: particular matter
PPI: protein–protein interaction

## 1 Introduction

Asthma is one of the most common inflammatory diseases that around 300 million people suffer from asthma globally, and the number is increasing to reach 400 million by 2025 (Dharmage et al. 2019). The causes of asthma include viral infection, exposure to environmental allergens, and air pollutants (Holgate et al. 2015). Along with modern industrialization and growing traffic‐related loads, air pollutants such as particulate matter (PM), carbon monoxide, and volatile organic compounds are considered putative environmental exposures that increased the prevalence of asthma (Khreis et al. 2017). These air pollutants exaggerated and developed asthma by inducing inflammatory mediator production and tissue remodeling such as smooth muscle hypertrophy, goblet cell hyperplasia, subepithelial fibrosis, and inflammatory cell infiltration in the lower airway (Takizawa 2015). Inflammatory reactions and remodeled tissue result in symptoms of asthma such as shortness of breath, chest tightness, wheezing, and coughing (Mims 2015). For that reason, medications widely used for relieving these symptoms are β2‐agonists and inhaled anti‐inflammatory corticosteroids (Heffler et al. 2018; Sobieraj et al. 2018). Despite recognized benefits, long‐term usage of these drugs has potential side effects such as immune‐suppressive effects or disturbance of endocrine function (Leung et al. 2017; Zdanowicz 2007). Therefore, there is a need to develop effective and safer therapeutic alternatives for asthma treatment.

Herbal medicines, extracted from plants and containing various organic chemicals, have a long history of relieving diverse symptoms including asthma (Wang et al. 2021). The anti‐asthmatic effects and safe long‐term usage of herbal medicines are supported by recent research (Kim et al. 2018; Shergis et al. 2016; Yang et al. 2022). Among diverse herbal medicines, essential oils extracted from aromatic plants by hydro‐distillation method contain volatile compounds and are widely used for relieving inflammatory symptoms (Bakkali et al. 2008). Recently, it is well known that volatile and lipophilic essential oils can reach the respiratory tract and effectively manage airway inflammation (Gandhi et al. 2020). Bergamot essential oil showed significant decreases in inflammatory cell number, thickness of bronchial airway walls, and collagen deposition in the airways in the ovalbumin (OVA)‐induced asthmatic mice (Feng et al. 2023). In addition, Safranal derived from *Crocus sativus* essential oil was reported to alleviate the asthmatic symptoms such as inflammatory cell infiltration, population of goblet cells, mucus secretion, and collagen deposition in the airways of OVA‐injected mice (Zhang et al. 2025). Furthermore, aerosolized essential oils are demonstrated to reach into the lungs directly, allowing for faster and more effective absorption of volatile bioactive compounds, without hepatic bypass (Martin and Finlay 2015; Rubin 2011). Regarding this, our previous study, aerosolized *Mentha piperita* L., *Asarum sieboldii* Miq., and *Abies holophylla* Maxim. essential oils have shown an anti‐asthmatic effect, respectively, utilizing a nebulizer to convert essential oils into fine particles for enhanced bioavailability of medication. *M*. *piperita* L. essential oil, as known as peppermint oil, ameliorated PM_10_‐induced asthma by regulating interleukin (IL)‐6/Janus kinase (JAK)2/Signal transducer and activator of transcription (STAT)3 signaling pathway (Kim et al. 2020). *A. sieboldii* Miq. essential oil exhibited anti‐fibrotic and anti‐inflammatory effects (Han et al. 2022). *A*. *holophylla* Maxim. leaf essential oil reduced the T helper (Th) 17‐mediated asthmatic symptoms (Park et al. 2022). From those results, we developed a newly formulated herbal formulation essential oil (FEO)‐03, which consists of *M*. *piperita* L., *A*. *sieboldii* Miq., and *A*. *holophylla* Maxim. essential oils for relieving asthma rather than using single herbs. It was predicted that inhalation of FEO‐03 can also ameliorate asthma, as constituents of FEO‐03 had anti‐asthmatic effects. Recently, network pharmacology presented a method for understanding herbal formulas and predicting potential targets for new herbal formulations (Tao et al. 2020). Because the “drug‐component‐target‐disease” approach of network pharmacology is an optimized integrative analytic technique to show the molecular actions of a new drug (Chen et al. 2025), it was conducted to predict potential targets of FEO‐03 on asthma in this study. Based on the network pharmacological prediction, in vivo experiments verified the regulatory effects of FEO‐03 inhalation on asthma by OVA and PM_10_‐induced asthmatic mice model.

## 2 Experimental

### 2.1 Network Construction and Functional Enrichment Analysis of Formulation Essential Oil

The network of FEO‐03 was constructed with compounds and co‐related genes of FEO‐03 ingredients. The compounds of FEO‐03 were organized based on the references (Table S1) (Lee and Hong 2009; Sun et al. 2014; Wu et al. 2012). Among 50, 10, and 31 compounds of each essential oil, 2 main compounds of each herb: *Menthol*, *Menthone*, *Methyl eugenol*, *Paeonal*, *Bicyclo[2.2.1]heptan‐2‐ol*, and *δ3‐Carene* were gathered (Table S2). These representative compounds were selected based on their relative abundance as identified by gas chromatographic‐mass spectrometry (GC–MS) profiling and key pharmacologically active constituents of *M*. *piperita* L., *A*. *sieboldii* Miq., and *A*. *holophylla* Maxim. essential oils, respectively, previously reported in the literature. The chemical‐gene co‐occurrence of each compound was collected through PubChem (https://pubchem.ncbi.nlm.nih.gov/) (Table S3). The co‐related targets of 6 main compounds were organized with the STRING database (https://string‐db.org/), using a Cytoscape STRING App and eliminating the duplicates; 272 targets were disclosed (Table S4). The asthma‐related genes were gathered using the GeneCards database (http://www.genecards.org/) by searching asthma as a keyword with the relevance score (Table S5). Relevance scores were obtained from the GeneCards database, where each gene is ranked by its association with asthma based on integrated evidence including text mining, functional annotations, and genomic data with the algorithm affected by Term Frequency‐Inverse Document Frequency principles, Annotation Type and Quality, and Integration of Multiple Data Sources. The overlapping genes of the FEO‐03 gene set and asthma gene set were counted, and genes with relevance scores exceeding 20 were sorted to prioritize genes with a stronger and more confident association with asthma (Table S6) (Gao et al. 2022). This threshold was selected based on a comprehensive review of similar network pharmacology studies utilizing GeneCards data, where scores typically range from 1 to over 100. Although there is no typically defined optimal threshold, a score of 20 or higher is commonly considered to indicate a robust association, helping to filter out less significant or spurious connections and enrich for biologically meaningful genes relevant to the disease context (Safran et al. 2010). In order to analyze the potential targets of FEO‐03, the functional enrichment analysis of the FEO‐03 gene network, including KEGG pathway enrichment, was conducted with Cytoscape (Table S7). The KEGG database was used to find the utilities and high‐level functions of biological systems. In the KEGG analysis, a *p* value < 0.01 was regarded as the cut‐off point (Lin and Hu 2021).

### 2.2 Preparation of Essential Oils for In Vivo Experiments

The dried leaves of *M*. *piperita* L. and dried rhizome of *A. sieboldii* Miq. were purchased from Dong‐Yang Herb Inc. (Seoul, Korea). The leaves of *A*. *holophylla* Maxim. were purchased from Gapyeong, Korea. In brief, 100 g herb of each species was placed in steam distillation apparatus with 1 L distilled water and distillation continued for 5 h at 100°C. The essential oil extract of each species was separated with centrifugation at 4000 rpm for 10 min and mixed by 4:2:3 ratios. The yields of FEO‐03 extracts were 1.02%. A voucher specimen of FEO‐03 (21‐01‐05‐KR‐200015) was deposited at the Department of Convergence Korean Medical Science, College of Korean Medicine, Kyung Hee University, Seoul, Korea at 4°C until use.

To identify the chemical compositions of each essential oil and FEO‐03 formulation, GC–MS analysis was performed. The used equipment was a GC‐2010 Shimadzu system combined with a GCMS‐QP2010 Plus quadrupole mass spectrometer (Agilent Technologies Inc., Santa Clara, CA, USA). Briefly, essential oil from *M*. *piperita* L. dissolved in dichloromethane was loaded on a 30‐m HP‐5MS capillary column at 45°C for 3 mins. The temperature of the injector was up to 250°C and held isothermally for 20 mins. *A. sieboldii* Miq. and *A*. *holophylla* Maxim. essential oils were injected on an Agilent DB‐5 column (30 m × 71 0.25 mm; 0.25 μm) encompassed a 100°C column oven temperature and a 130°C injection temperature. The chemical compositions of the oils are presented (Figure S1, Table S8).

### 2.3 In Vivo Mice Models of OVA and PM_10_ ‐Induced Asthma

The 5‐week‐old female BALB/c mice were purchased from DBL Co. (Eumseong, Korea). Mice were acclimatized in a SPF conditioned room (12 h/12 h light/dark cycle, 22°C ± 2°C temperature, 55% ± 10% humidity) for 1 week. All processes of the animal experiment were approved by the Institutional Animal Ethics Committee of Kyung Hee University in Korea (KHUASP(SE)‐20‐363; Seoul, Korea). After accumulation, mice were randomly divided into five groups (*n* = 7); (1) NOR, the normal saline‐sensitized group, (2) OVA + PM_10_, the ovalbumin and PM_10_‐sensitized group, (3) DEX, dexamethasone treated group, (4) FEO‐03 0.0009, FEO‐03 0.0009% v/v treated group, and (5) FEO‐03 0.09%, FEO‐03 0.09% v/v treated group. We placed all mice on a large bench and randomly assigned one to each group to ensure that potential confounding factors were evenly distributed across all groups. After randomization, each mouse was marked with numbers and distinguished. The sample size (*n* = 7) was determined based on the pilot study and previous studies that were similar to animal experimental design compared to our study (Kim et al. 2020; Wu et al. 2020). On Days 0, 21, 28, and 35, all experimental groups except the NOR group were injected intraperitoneally with 0.1 mL saline containing 1 mg ovalbumin (OVA) emulsified in 500 μg aluminum hydroxide for first sensitization; the NOR group was injected intraperitoneally with 0.1 mL saline. On Days 42–44, all experimental groups except the NOR group received intranasal instillation of 1 mg OVA and 100 μg PM_10_ in 50 μL saline. From Days 1 to 47, the DEX, FEO‐03 0.0009%, and FEO‐03 0.09% groups inhaled aerosolized DEX and FEO‐03 by nebulizer (Philips, Amsterdam, Netherlands), 3 times per week and 5 min per time. The spray amount was 1 mL/min and the concentration of DEX was 0.06% w/v (2 mg/kg), as those of FEO‐03 0.0009% and 0.09% were 0.0009% v/v and 0.09% v/v of FEO‐03 in saline. The NOR and OVA + PM_10_ groups inhaled saline. All mice were sacrificed on day 49. Mice were euthanized by intraperitoneal injection of a mixture of alfaxalone (80 mg/kg) and xylazine (10 mg/kg) in a final volume of 100 μL per mouse, followed by cervical dislocation. This procedure was performed in accordance with institutional animal ethics guidelines and approved protocols. No animals or data points were excluded from the study, and all animals were included in the final analysis.

### 2.4 Histological Analysis

The right lung tissue and trachea were dissected and fixed with 10% formalin. After dehydration through a graded ethanol series (70%–100%), tissues were cleared in xylene and embedded in paraffin. The embedded tissues were sectioned at 5 μm thickness using a rotary microtome. The sliced specimens were stained with hematoxylin and eosin (H&E) for evaluating epithelial thickness, then stained with a periodic acid‐Schiff (PAS) kit (Abcam plc., Cambridge, UK) for assessing hyperplasia of goblet cells. The lung tissues were additionally stained with Masson's trichrome stain kit (Polysciences Inc., Warrington, PA, USA) for evaluation of lung fibrosis and collagen accumulation, and the fibrotic area (%) was measured in five random fields per section at 200× magnification.

### 2.5 Broncho Alveolar Lavage Fluid (BALF) Analysis

After cervical dislocation, tracheostomy was performed and a polyethylene catheter (0.8 mm ID) was inserted into the trachea. The lungs were lavaged gently with 1 mL of cold phosphate‐buffered saline (PBS) using a syringe. The lavage was performed once, and the recovered BALF (~0.8–0.9 mL) was collected into microtubes on ice. The BALF was centrifuged at 1200 rpm for 10 min at 4°C, and the resulting cell pellet was resuspended in 1 mL PBS and centrifuged again under the same conditions. The BALF cells were stained with Wright‐Giemsa stain (Sigma‐Aldrich, USA) for 10 min, and the total number of inflammatory cells, macrophages, eosinophils, and neutrophils was counted using a hemocytometer under a light microscope (200× magnification). At least five random fields per sample were counted to calculate mean cell numbers.

### 2.6 Enzyme‐Linked Immunosorbent Assay (ELISA)

Blood samples were collected via retro‐orbital bleeding under light anesthesia using K2‐EDTA‐coated vacutainers (BD, Franklin Lakes, NJ, USA). Plasma was separated by centrifugation at 1200 rpm for 30 min at 4°C, and the supernatant serum was transferred to clean microcentrifuge tubes and stored at −80°C until analysis. Serum levels of immunoglobulin E (IgE) and IgG2a were quantified using commercial ELISA kits (BD Biosciences, San Diego, CA, USA) according to the manufacturer's instructions. Absorbance was measured at 450 nm using a microplate reader (BioTek Epoch), and concentrations were calculated based on standard curves.

### 2.7 RNA Extraction for RT‐PCR Analysis

Total RNA was extracted from lung tissue samples (30 mg per sample) using 1 mL of TRIzol reagent (Invitrogen, Carlsbad, CA, USA) according to the manufacturer's protocol. RNA of each group was extracted by combining the lung tissues of all 7 mice per group. RNA purity and concentration were assessed using a NanoDrop spectrophotometer (Thermo Fisher) based on the A260/A280 ratio, and 1000 ng of total RNA was used for cDNA synthesis using the Maxime RT premix kit (iNtRON Biotechnology, Korea). Reverse transcription was performed at 45°C for 60 min, followed by 95°C for 5 min in a thermal cycler (Bio‐Rad T100). PCR amplification was carried out using the Maxime PCR premix under the following cycling conditions: 95°C for 30 s, 58°C for 30 s, and 72°C for 30 s, for a total of 35 cycles. Primer sequences and accession numbers for each target gene are listed to ensure specificity and reproducibility (Table S9). PCR products were resolved by electrophoresis in 2% agarose gel containing ethidium bromide and visualized under UV illumination. The relative band intensities were quantified using ImageJ software, and gene expression levels were normalized to Gapdh as a housekeeping gene.

### 2.8 Western Blotting

The lung tissues of each group (*n* = 7 without exclusion) were lysed with T‐PER tissue protein extraction reagent (Thermo scientific, Rockford, IL, USA) containing protease inhibitor (Roche, Hoffmann, USA) in order to extract protein. The 10 μg of protein samples were separated by sodium dodecyl sulfate–polyacrylamide gels. Then, the protein samples were transferred to polyvinylidene difluoride (PVDF) membranes. The membranes were blocked by 3% bovine serum albumin (BSA) for 1 h and incubated overnight with the primary antibodies (1:1000; Cell signaling, USA) at 4°C. Then, the membrane was incubated using horseradish peroxidase (HRP)‐conjugated secondary antibodies (1:3000; Cell signaling, USA) for 1 h at room temperature. Detailed antibody information, including source, catalog number, dilution, and loading control, is provided (Table S10). The blots were detected using Asherman Imagequant 800 (Amersham Pharmacia Biotech, Uppsala, Sweden).

### 2.9 Statistical Analysis

All data generated from this study was included for statistical analysis without any trimming. Equal sample sizes (*n* = 7) were maintained across experiments to support statistical robustness. Statistical analysis was performed by an independent individual who did not participate in the experimental procedures or data analysis with the random group codes. One‐way analysis of variance (ANOVA) was used to compare differences among groups, followed by Tukey's post hoc test for multiple comparisons (GraphPad Software Inc., La Jolla, CA, USA). All data are expressed as the mean ± standard error of the mean (SEM), and statistical significance was defined as *p* < 0.05.

## 3 Results

### 3.1 Gene Comparison and Functional Enrichment Analysis of FEO‐03 Network

**FIGURE 1:**
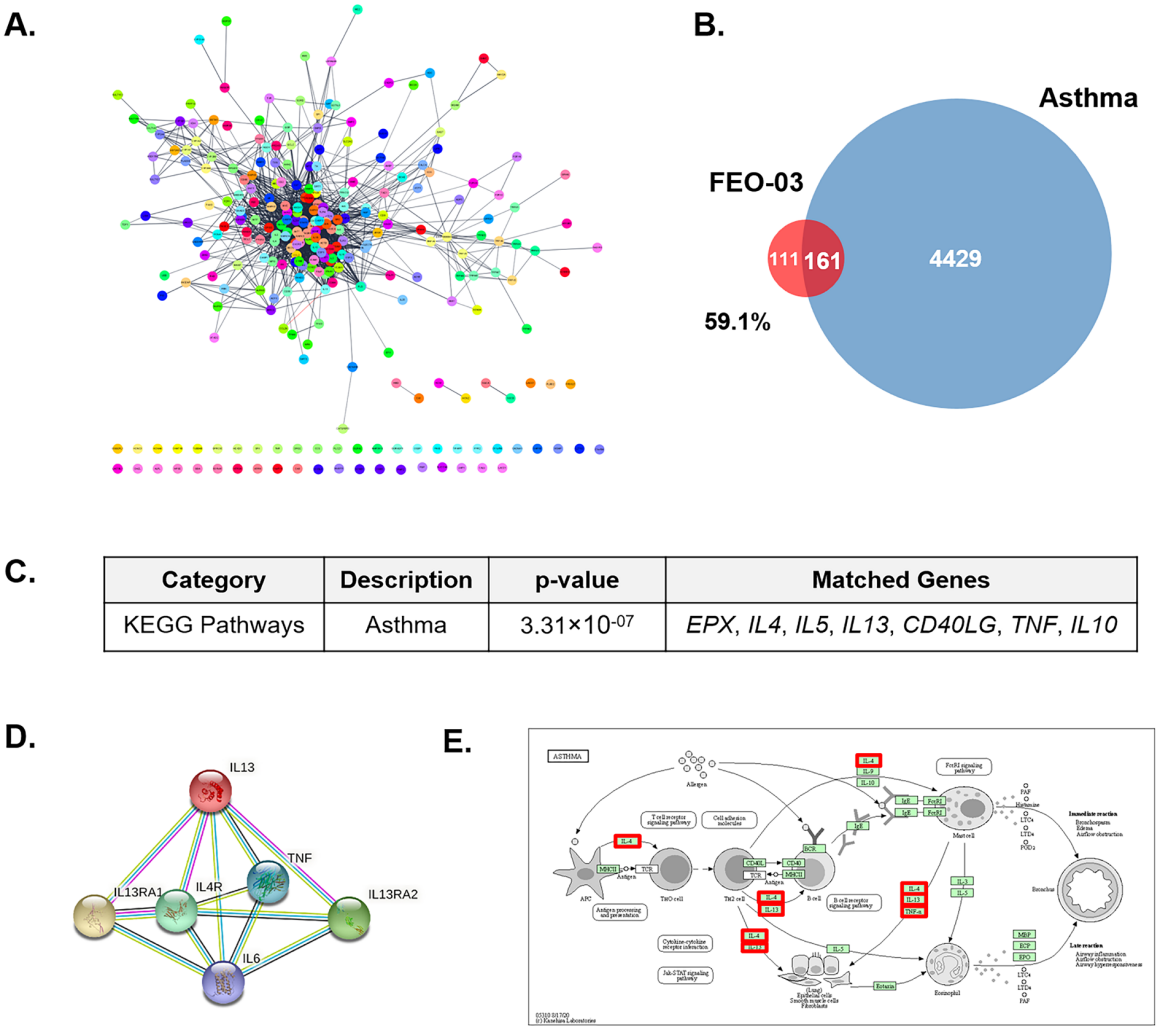
The prediction of FEO‐03 essential oil formulation on asthma by network pharmacology analysis. The PPI network of FEO‐03 essential oil formulation (A). The Venn diagram of genes related both to FEO‐03 and asthma, showing the relevant overlapped genes (B). The target prediction of FEO‐03 on KEGG Pathways databases performed by functional enrichment analysis (C). The STRING network of IL‐13 (D). Asthma KEGG pathway enrichment analysis. The red box represents the target genes in our study (E).

The FEO‐03 network constructing compounds *Menthol*, *Menthone*, *Methyl eugenol*, *Paeonal*, *Bicyclo[2.2.1]heptan‐2‐ol*, and *δ3‐Carene* had 100, 100, 99, 100, 7, and 55 co‐efficient genes, respectively. The protein–protein interaction (PPI) network, composed of 272 nodes with 1689 edges, displayed the targets of FEO‐03 and the connectivity between targets (Figure 1A). The overlapping genes between the asthma gene set and FEO‐03 network were 161, occupying 59.1% of FEO‐03 network (Figure 1B). The 7 relevant overlapping genes with relevance scores > 20 were *IL13*, *TNF*, *IL3*, *IL5*, *CXCL8*, *TSLP*, and *IL10*. The functional enrichment analysis predicting human‐related mechanisms of FEO‐03 was performed (Table S6). The relationship between the FEO‐03 network and the KEGG asthma pathway was confirmed, and matched genes were identified. The relevance between the FEO‐03 network and asthma on the KEGG Pathways database presented *EPX*, *IL4*, *IL5*, *IL13*, *CD40LG*, *TNF*, and *IL10* according to *p* value (Figure 1C). The common gene of the FEO‐03 network and asthma with the highest relevance score was *IL13*. In addition, the STRING network of *IL13* visualized protein–protein interaction and functional partners (Figure 1D). The targets of the FEO‐03 network, IL13, and functional partners *IL13RA1*, *IL13RA2*, *IL4R*, *IL6*, and *TNF* were presented in a red box from the asthma diagram of KEGG Pathways (Figure 1E).

### 3.2 Histological Changes of Respiratory Tracts in OVA and PM_10_‐Exposed Mice

**FIGURE 2:**
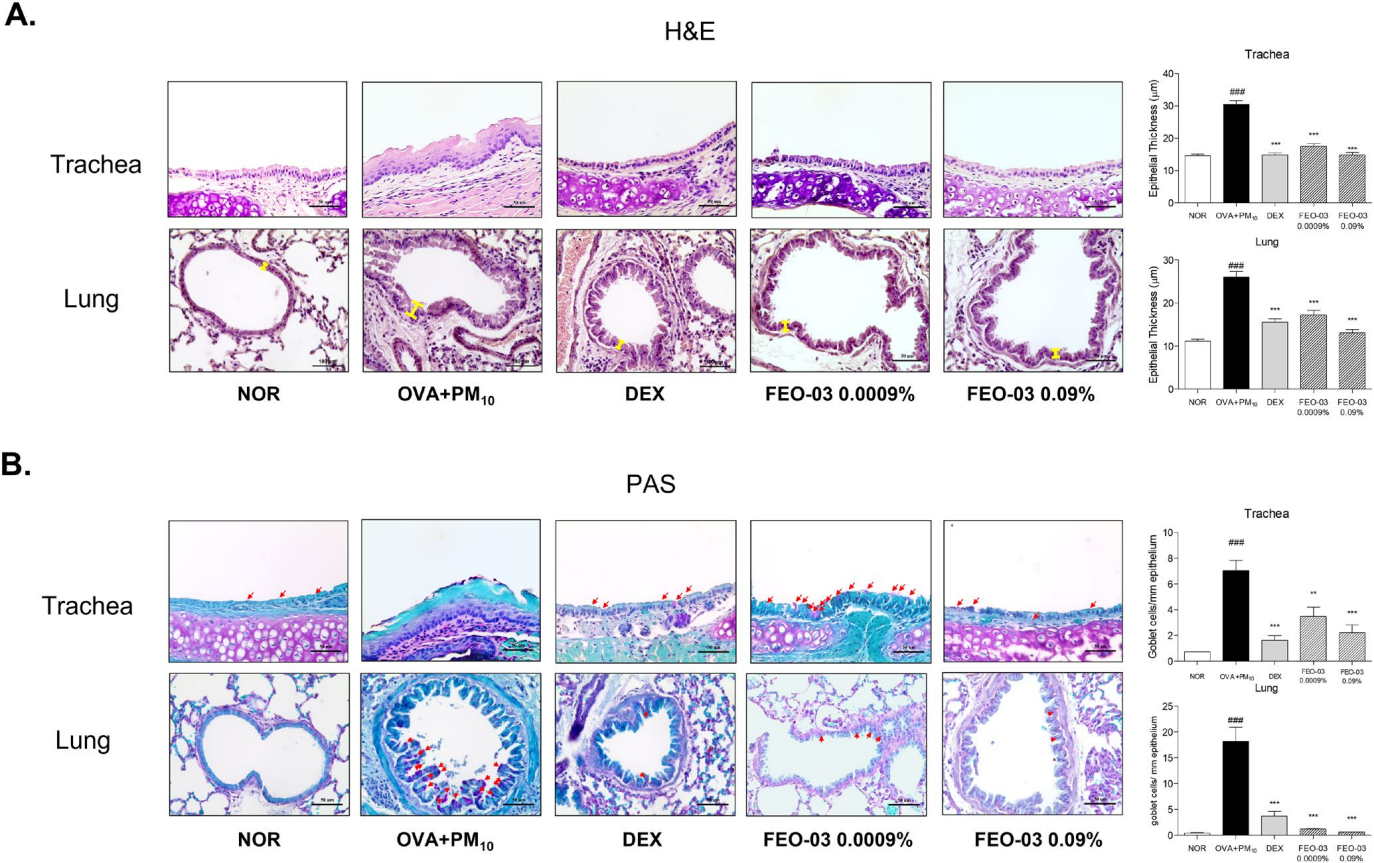
Histological changes in lung and trachea tissues of particulate matter 10 (PM_10_)‐induced mice. Representative images of hematoxylin and eosin (H&E) staining measuring epithelial thickness (A). Periodic acid–Schiff (PAS) staining for counting goblet cells. Red arrows: Goblet cell (B). Scale bar = 50 μm. Statistical results are presented as the mean ± SEM (*n* = 7). ^###^ *p* < 0.001 vs. NOR group; *p < 0.05, **p < 0.01 and ***p < 0.001 vs. OVA + PM10 group.

H&E staining of trachea and lung tissue was performed, and epithelial thickness was measured in order to evaluate inflammatory cell infiltration. The thickness of pulmonary epithelium of trachea and lung tissues was increased by 109.8% and 144.7% in the OVA + PM_10_ treated group, respectively, compared to normal mice. 0.0009% and 0.09% FEO‐03 treatment significantly reduced tracheal epithelium thickness by 42.5% and 51.2% compared to the OVA + PM_10_‐treated group. Also, the pulmonary epithelium thickness in the lung tissues was decreased by 34% and 49.8%, respectively (Figure 2A). PAS staining was conducted for evaluating hyperplasia of goblet cells in trachea and lung epithelium. The OVA + PM_10_‐treated group showed 9.57 times and 41.56 times increase in the number of goblet cells of trachea and lung, respectively. The FEO‐03 0.0009% and 0.09% treatment reduced tracheal goblet cell numbers by 50.7% and 68.8%. Additionally, pulmonary goblet cell counts in the lung tissues were decreased by 93.4% and 96.2% by FEO‐03 treatments (Figure 2B).

### 3.3 Modulation of Inflammatory Responses in OVA and PM_10_‐Exposed Mice

**FIGURE 3:**
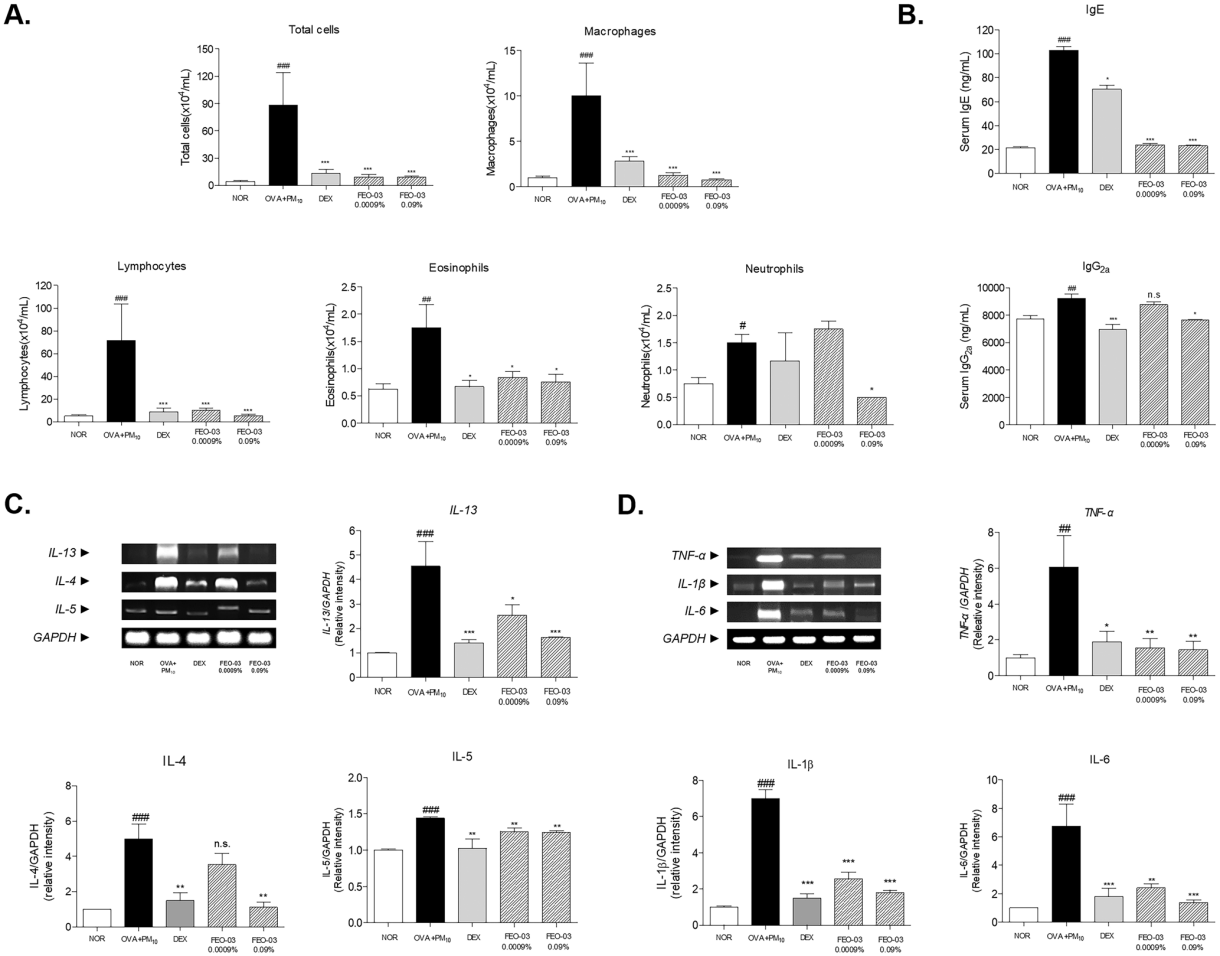
Anti‐inflammatory effects of FEO‐03 essential oil formulation in OVA + PM_10_‐induced asthmatic mice. The number of total cells, macrophages, neutrophils, eosinophils, and neutrophils in bronchoalveolar lavage fluid (BALF) (A). The serum IgE and IgG2a expression of OVA + PM_10_ ‐induced mice (B). The mRNA expression of Th2‐specific cytokines (C) and pro‐inflammatory cytokines (D) in the lung tissues. The results were presented as the mean ± SEM (*n* = 7). ^###^ *p* < 0.001 vs. NOR group; *p < 0.05, **p < 0.01 and ***p < 0.001 vs. OVA + PM_10_ group. IgE, Immunoglobulin E; IgG2a, Immunoglobulin G2a; IL, interleukin; TNF‐α, tumor necrosis factor‐alpha.

The number of total cells, macrophages, lymphocytes, eosinophils, and neutrophils was counted in BALF. The sensitization with the OVA and PM_10_ increased the number of total cells. Sensitization with OVA and PM_10_ resulted in a significant inflammatory response in the lungs, as shown by a nearly 6‐fold increase in total cell number in BALF compared to the normal group. Inhalation of 0.09% FEO‐03 markedly suppressed this cellular influx, reducing the total cell count by 75.0% relative to the OVA + PM_10_ group and bringing it close to normal levels (Figure 3A). Similar trends were observed in the reduction of macrophages, lymphocytes, eosinophils, and neutrophils compared to the OVA + PM_10_ group, with levels approaching those seen in the NOR group. The level of IgE and IgG2a measured from the serum of asthmatic mice was significantly increased. The treatment of FEO‐03 in a concentration of 0.09% significantly decreased the levels of IgE and IgG2a by 77.38% and 17.18%, compared to the OVA + PM_10_ group (Figure 3B).

The Th2‐specific cytokines, *IL‐13*, *IL‐4*, and *IL‐5* mRNA expression in OVA + PM_10_ exposed groups. Treatment with 0.0009% and 0.09% FEO‐03 reduced *IL‐13* expression by 43.95% and 64.07%, respectively. Similarly, *IL‐4* expressions were decreased by 28.92% and 77.74%, and *IL‐5* expression was decreased by 12.89% and 13.73%, respectively, following FEO‐03 treatment. The FEO‐03 0.0009% and 0.09% treated group showed a 28.92% and 77.74% decrease in expressions of *IL‐4*. In addition, the *IL‐5* expressions were dropped by 12.89% and 13.73% following FEO‐03 0.0009% and 0.09% treatment (Figure 3C). In addition, treatment with FEO‐03 0.0009% and 0.09% decreased the expression of *TNF‐a* by 74.18% and 76.14%. The FEO‐03 0.0009% and 0.09% treatment significantly decreased the mRNA expressions of *IL‐1β* by 63.56% and 74.4%. Also, the *IL‐6* expression was reduced by 64.25% and 79.47%, respectively (Figure 3D).

### 3.4 Deposition of Fibrosis in the Lung Tissues of OVA and PM_10_ ‐Exposed Mice

**FIGURE 4:**
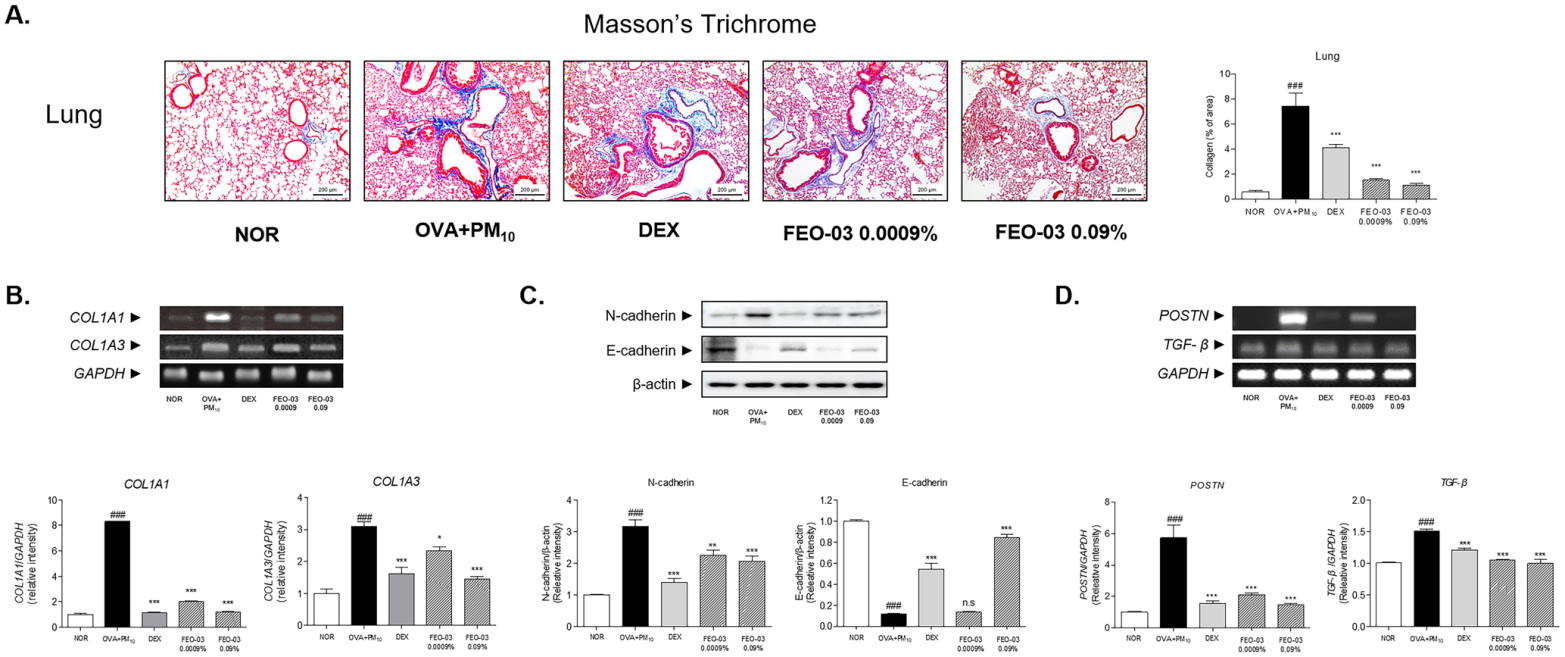
Inhibitory effects of FEO‐03 essential oil formulation on collagen deposition in OVA + PM_10_‐induced asthmatic mice. Masson's trichrome staining for measuring the amount of collagen deposition (A). The mRNA expression of fibril forming mediators *COL1A1* and *COL1A3* (B), the protein expression of N‐cadherin and E cadherin fibrotic mediators (C), and *POSTN* and *TGF‐1β* (D) in lung tissues. The results were presented as the mean ± SEM (*n* = 7). ^###^ *p* < 0.001 vs. NOR group; *p < 0.05, **p < 0.01, and ***p < 0.001 vs. OVA + PM_10_ group. *COL1A1*, collagen, type I, alpha 1; *COL1A3*, collagen, type I, alpha 3; *POSTN*, periostin; TGF‐β, transforming growth factor beta.

The collagen deposition in lung tissues was observed by Masson's Trichrome staining. The OVA + PM_10_ treated group showed an increased area of collagen in lung epithelial tissue. The FEO‐03 0.0009% and 0.09% treatment significantly decreased the blue‐stained collagen area by 79.4% and 85.2%, respectively (Figure 4A). The expression levels of *COL1A1* were down‐regulated in the FEO‐03 0.0009% and 0.09% treated group by 75.99% and 85.77%. The *COL1A3* expression was 24.35% and 53.1% compared to the PM_10_ + OVA treated lung tissues (Figure 4B). Moreover, the N‐cadherin and E‐cadherin expressions in the OVA + PM_10_‐exposed lung tissue were increased by 215.72% and decreased by 88.32%, respectively, compared to normal lung tissues. The treatment of FEO‐03 at the 0.0009% and 0.09% concentrations significantly down‐regulated the protein expression of N‐cadherin by 28.85% and 34.56%, whereas the expression levels of E‐cadherin were increased in the 0.0009% and 0.09% FEO‐03 treated lung tissues by 622.01% (Figure 4C).

In terms of periostin‐TGF‐β signaling pathway, OVA and PM_10_ sensitization showed an increase in mRNA expressions of *POSTN* and *TGF‐β* by 474.1% and 49.9%, respectively (Figure 4D). The treatment of FEO‐03 0.0009% and 0.09% significantly decreased the *POSTN* levels in PM_10_ + OVA exposed lung tissues by 63.3% and 74.66%. The decrease rate of *TGF‐β* expressions was 30.51% and 33.71% in the aerosolized FEO‐03 groups.

## 4 Discussion

Inflammation and structural remodeling of airway epithelial cells are major responses in asthma (Hammad and Lambrecht 2021). Especially, several studies have reported that exposure to air pollution causes an increase in airway wall thickness and epithelial goblet cell hyperplasia, further leading to lung fibrosis in allergic asthmatic patients (Misiukiewicz‐Stepien and Paplinska‐Goryca 2021). Considering the limitations of current treatments for asthma and the reality of progressively worsening air pollution, therapeutics, which have advantages in the treatment of asthma without adverse effects, are needed to develop. FEO‐03 is a newly developed essential oil formulation for the purpose of reduction of airway inflammation and remodeling through inhalation, reaching the terminal bronchioles and entering the alveoli. In the present study, we aimed to determine whether the inhalation of FEO‐03 by a nebulizer could effectively ameliorate the asthmatic symptoms with the inhibition of airway inflammation and remodeling. Prior to the experimental study, the potential efficacy and functional mechanisms of FEO‐03 essential oil were predicted by a network pharmacology analysis. The close association of the FEO‐03 network and asthma gene set was revealed by finding 59.1% overlapping target genes among all genes of FEO‐03 with asthma‐targeted genes. The potential targets of FEO‐03 on asthma that resulted from a functional enrichment analysis were Th2‐specific cytokines and pro‐inflammatory cytokines. The most correlated targets of the FEO‐03 network on asthma were IL‐13, IL‐4, and IL‐5. Based on the network pharmacological results, FEO‐03 essential oil was inhaled by a nebulizer in OVA and PM_10_‐exposed asthmatic mice. Consistent with network investigations, we found that inhalation of FEO‐03 through a nebulizer decreased the thickness of the pulmonary epithelium and the proportion of goblet cells in the OVA + PM_10_‐exposed upper respiratory system, such as the trachea and lung tissues. The FEO‐03 treatment normalized the structural remodeling in airway epithelial cells.

Allergic asthmatic responses triggered by dust and pollen are characterized by Th2 cell responses, which can be detected in atopic dermatitis and allergic rhinitis (Wang et al. 2023). Upon sensitizing environmental allergens, Th2‐specific cytokines such as IL‐4, IL‐5, and IL‐13 provoke the accumulation of eosinophils in the airway epithelial cells, mucus overproduction, and IgE synthesis. These inflammatory responses eventually trigger bronchial hyperresponsiveness, goblet cell maturation, and airway tissue remodeling (Leon and Ballesteros‐Tato 2021). In addition, it is now clear that acute pro‐inflammatory cytokines, including TNF‐α, IL‐1β and IL‐6, in exposure to ambient PM exert their immunomodulatory effects in the lung, contributing to the initiation of inflammation (Yang et al. 2017). For that reason, control of underlying inflammatory responses with Th2 cytokines and other mediators is the primary objective of asthma treatment to inhibit the development of disease progression (Holgate and Polosa 2008). In this study, the proportion of inflammatory cells and level of immunoglobulins were determined in BALF and serum, respectively. FEO‐03 inhalation significantly reduced the numbers of inflammatory cells, including macrophages, lymphocytes, eosinophils, and neutrophils in BALF, and serum IgE levels in OVA + PM_10_‐exposed mice, indicating that FEO‐03 effectively inhibited airway inflammation in a condition of asthma. Also, FEO‐03 treatment significantly reduced the production of Th2‐related cytokines, *IL‐13*, *IL‐4*, and *IL‐5*, along with innate‐response related cytokines, *TNF‐α*, *IL‐1β* and *IL‐6*, in OVA + PM_10_‐exposed mice lung tissues. Taken together, FEO‐03 inhibited the proliferation of inflammatory cells in BALF and serum IgE by regulating the production of Th2‐related cytokines and innate‐response related cytokines. Inconsistent with the histological data, FEO‐03 could effectively attenuate the asthmatic symptoms mediated by Th2‐related cytokines and innate‐response related cytokines. Because allergic asthmatic patients are commonly characterized by the histological alteration including pulmonary epithelium thickening and increase of neutrophils in BALF with the Th2 specific cytokines, which are primary outcomes from our study, we guess that FEO‐03 might be useful for treating asthma.

In the progression of disease, excessive airway remodeling accompanied by subepithelial fibrosis and collagen deposition in the airway wall appears. Although collagen type I and III are well‐known fibrillar components of the normal matrix, which supports the airway wall mechanically, the airway wall of asthmatic patients who are exposed to repeated allergens manifests abnormal expansion and deposition of collagen in the bronchial submucosa (Palmans et al. 2002). Type 1 collagen components COL1A1 and COL1A3 are fibrotic markers produced by fibroblasts, and the deposition of collagen is closely controlled by the expression of COL1A1 and COL1A3 proportionately (Poulalhon et al. 2006). Therefore, this fibrotic airway remodeling leads to irreversible respiratory difficulties and exacerbation of airway hyperresponsiveness (Yamauchi and Inoue 2007). In particular, epithelial‐to‐mesenchymal transition (EMT) plays an aggravating role in subepithelial fibrosis in the development of asthma (Pain et al. 2014). Among them, periostin (gene name: *POSTN*) is a matricellular protein that is involved in diverse inflammatory responses in asthma, including the development of the Th2 phenotype (Li et al. 2015). IL‐4 and IL‐13, together with TGF‐β, stimulate the production of periostin, which then promotes fibroblast differentiation and EMT in asthma (Mitamura et al. 2018). In the OVX and PM_10_‐exposed lung tissues, there was severe collagen deposition stained by blue dye. In addition, the mRNA levels of *COL1A1* and *COL1A3* with the cadherin switching were markedly increased in the lung tissues of asthmatic mice. FEO‐03 treatment significantly inhibited the expressions of *COL1A1* and *COL1A3*. Additionally, FEO‐03 treatment significantly regulated the cadherin switching, which are important adhesion molecules for maintaining epithelial integrity as well as the EMT (Loh et al. 2019). Moreover, the periostin and *TGF‐β* mRNA expressions were significantly decreased in the asthmatic lung tissues by FEO‐03 inhalation. Along with the results from the inhibition of Th2‐specific cytokines by FEO‐03, it was assumed that FEO‐03 could be effective in maintaining epithelial integrity by regulating adhesion molecules.

**FIGURE 5:**
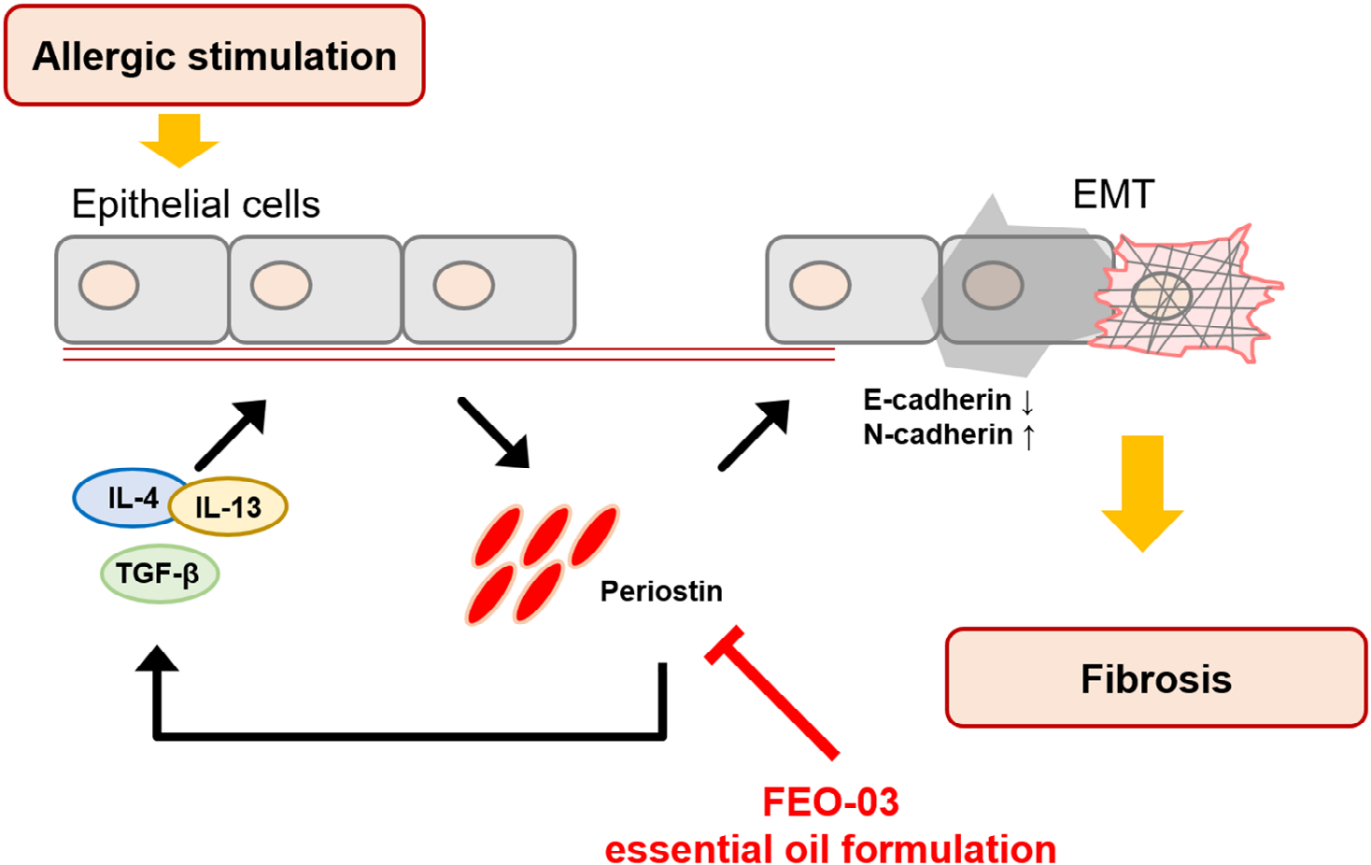
Diagram of potential mechanisms of FEO‐03 essential oil formulation for asthma. FEO‐03 consisting of *Mentha piperita* L., *Asarum sieboldii* Miq., and *Abies holophylla* Maxim. essential oils significantly inhibited the periostin, a matricellular protein to induce the Th2‐specific cytokines such as IL‐4 and IL‐13 and promote cadherin switching for epithelial‐to‐mesenchymal transition in asthma.

To sum up, this study suggests that FEO‐03 could alleviate asthma through regulating inflammatory and fibrotic responses. First, the beneficial effects of FEO‐03 on asthma were demonstrated based on network pharmacological analysis. The further study investigated the anti‐asthmatic effects of FEO‐03 inhalation on an OVA + PM_10_‐induced mice model. The results showed that FEO‐03 reduced inflammatory and fibrotic responses in airway tissues by downregulating the production of Th2 cell associated factors, IL‐13 and IL‐4, which were identified as potential targets of FEO‐03 through network pharmacology. The study also found that FEO‐03 reduced the expression of fibrotic markers through the periostin/TGF‐β signaling pathway in asthma (Figure 5). These findings suggest that FEO‐03 may be a promising therapeutic agent for asthma treatment. Nevertheless, there are still limitations to consider in this study. In terms of network pharmacology analysis, target prediction based on public databases may be limited for compounds with sparse chemical‐gene interaction records, although we meticulously collected all currently accessible databases for the compounds identified in *M. piperita* L., *A. sieboldii* Miq., and *A. holophylla* Maxim. essential oils. This reflects a broader challenge in network pharmacology applications for natural products. Additionally, it is limited by the lack of long‐term safety evaluations and clinical validation. Future studies should investigate the chronic effects and clinical applicability of FEO‐03 in human populations.

## Author Contributions

**Mi Hye Kim:** data curation (equal), formal analysis (lead), investigation (lead), writing – original draft (lead), writing – review and editing (lead). **Seong Chul Jin:** data curation (equal), formal analysis (equal), investigation (supporting), methodology (lead), writing – review and editing (supporting). **Woong Mo Yang:** conceptualization (lead), funding acquisition (lead), methodology (supporting), project administration (lead), supervision (lead), writing – original draft (supporting), writing – review and editing (supporting).

## Conflicts of Interest

The authors declare no conflicts of interest.

## Supporting information


**Figure S1:** fsn370763‐sup‐0001‐FigureS1.tif.

**Table S1:** fsn370763‐sup‐0002‐TablesS1‐S10.zip.
**Table S2:** fsn370763‐sup‐0002‐TablesS1‐S10.zip.
**Table S3:** fsn370763‐sup‐0002‐TablesS1‐S10.zip.
**Table S4:** fsn370763‐sup‐0002‐TablesS1‐S10.zip.
**Table S5:** fsn370763‐sup‐0002‐TablesS1‐S10.zip.
**Table S6:** fsn370763‐sup‐0002‐TablesS1‐S10.zip.
**Table S7:** fsn370763‐sup‐0002‐TablesS1‐S10.zip.
**Table S8:** fsn370763‐sup‐0002‐TablesS1‐S10.zip.
**Table S9:** fsn370763‐sup‐0002‐TablesS1‐S10.zip.
**Table S10:** fsn370763‐sup‐0002‐TablesS1‐S10.zip.

## Data Availability

The data supporting this article have been included as part of the [Link], [Link].

## References

[fsn370763-bib-0001] Bakkali, F. , S. Averbeck , D. Averbeck , and M. Idaomar . 2008. “Biological Effects of Essential Oils—A Review.” Food and Chemical Toxicology 46, no. 2: 446–475. 10.1016/j.fct.2007.09.106. 17996351

[fsn370763-bib-0002] Chen, Y. , S. Yang , H. Shi , J. Cui , and Z. Li . 2025. “Molecular Mechanisms Underlying Apple Extract Ameliorates Depression‐Incident Cognitive Dysfunction Based on Network Pharmacology.” Food Science & Nutrition 13, no. 6: e70408. 10.1002/fsn3.70408. 40491976 PMC12146215

[fsn370763-bib-0003] Dharmage, S. C. , J. L. Perret , and A. Custovic . 2019. “Epidemiology of Asthma in Children and Adults.” Frontiers in Pediatrics 7: 246. 10.3389/fped.2019.00246. 31275909 PMC6591438

[fsn370763-bib-0004] Feng, S. , G. Xu , Y. Fu , Q. Ding , and Y. Shi . 2023. “Exploring the Mechanism of Bergamot Essential Oil Against Asthma Based on Network Pharmacology and Experimental Verification.” ACS Omega 8, no. 11: 10202–10213. 10.1021/acsomega.2c07366. 36969419 PMC10034984

[fsn370763-bib-0005] Gandhi, G. R. , A. B. S. Vasconcelos , G. H. Haran , et al. 2020. “Essential Oils and Its Bioactive Compounds Modulating Cytokines: A Systematic Review on Anti‐Asthmatic and Immunomodulatory Properties.” Phytomedicine 73: 152854. 10.1016/j.phymed.2019.152854. 31036393

[fsn370763-bib-0006] Gao, Q. , W. Zhang , T. Li , et al. 2022. “Interrelationship Between 2019‐nCov Receptor DPP4 and Diabetes Mellitus Targets Based on Protein Interaction Network.” Scientific Reports 12, no. 1: 188. 10.1038/s41598-021-03912-6. 34996987 PMC8741798

[fsn370763-bib-0007] Hammad, H. , and B. N. Lambrecht . 2021. “The Basic Immunology of Asthma.” Cell 184, no. 6: 1469–1485. 10.1016/j.cell.2021.02.016. 33711259

[fsn370763-bib-0008] Han, J. M. , M. H. Kim , Y. Choi , G. Kim , and W. M. Yang . 2022. “Exploring the Potential Effects and Mechanisms of *Asarum sieboldii* Radix Essential Oil for Treatment of Asthma.” Pharmaceutics 14, no. 3: 558. 10.3390/pharmaceutics14030558. 35335934 PMC8953372

[fsn370763-bib-0009] Heffler, E. , L. N. G. Madeira , M. Ferrando , et al. 2018. “Inhaled Corticosteroids Safety and Adverse Effects in Patients With Asthma.” Journal of Allergy and Clinical Immunology: In Practice 6, no. 3: 776–781. 10.1016/j.jaip.2018.01.025. 29408385

[fsn370763-bib-0010] Holgate, S. T. , and R. Polosa . 2008. “Treatment Strategies for Allergy and Asthma.” Nature Reviews. Immunology 8, no. 3: 218–230. 10.1038/nri2262. 18274559

[fsn370763-bib-0011] Holgate, S. T. , S. Wenzel , D. S. Postma , S. T. Weiss , H. Renz , and P. D. Sly . 2015. “Asthma.” Nature Reviews Disease Primers 1, no. 1: 15025. 10.1038/nrdp.2015.25. PMC7096989 27189668

[fsn370763-bib-0012] Khreis, H. , C. Kelly , J. Tate , R. Parslow , K. Lucas , and M. Nieuwenhuijsen . 2017. “Exposure to Traffic‐Related Air Pollution and Risk of Development of Childhood Asthma: A Systematic Review and Meta‐Analysis.” Environment International 100: 1–31. 10.1016/j.envint.2016.11.012. 27881237

[fsn370763-bib-0013] Kim, J. H. , G. Kismali , and S. C. Gupta . 2018. “Natural Products for the Prevention and Treatment of Chronic Inflammatory Diseases: Integrating Traditional Medicine Into Modern Chronic Diseases Care.” Evidence‐Based Complementary and Alternative Medicine 2018: 9837863. 10.1155/2018/9837863. 29805468 PMC5899861

[fsn370763-bib-0014] Kim, M. H. , S. J. Park , and W. M. Yang . 2020. “Inhalation of Essential Oil From *Mentha piperita* Ameliorates PM_10_‐Exposed Asthma by Targeting IL‐6/JAK2/STAT3 Pathway Based on a Network Pharmacological Analysis.” Pharmaceuticals (Basel) 14, no. 1: 2. 10.3390/ph14010002. 33374928 PMC7821947

[fsn370763-bib-0015] Lee, J. H. , and S. K. Hong . 2009. “Comparative Analysis of Chemical Compositions and Antimicrobial Activities of Essential Oils From *Abies holophylla* and *Abies koreana* .” Journal of Microbiology and Biotechnology 19, no. 4: 372–377. 10.4014/jmb.0811.630. 19420993

[fsn370763-bib-0016] Leon, B. , and A. Ballesteros‐Tato . 2021. “Modulating Th2 Cell Immunity for the Treatment of Asthma.” Frontiers in Immunology 12: 637948. 10.3389/fimmu.2021.637948. 33643321 PMC7902894

[fsn370763-bib-0017] Leung, J. S. , D. W. Johnson , A. J. Sperou , et al. 2017. “A Systematic Review of Adverse Drug Events Associated With Administration of Common Asthma Medications in Children.” PLoS One 12, no. 8: e0182738. 10.1371/journal.pone.0182738. 28793336 PMC5549998

[fsn370763-bib-0018] Li, W. , P. Gao , Y. Zhi , et al. 2015. “Periostin: Its Role in Asthma and Its Potential as a Diagnostic or Therapeutic Target.” Respiratory Research 16, no. 1: 57. 10.1186/s12931-015-0218-2. 25981515 PMC4437675

[fsn370763-bib-0019] Lin, Y. , and Z. Hu . 2021. “Bioinformatics Analysis of Candidate Genes Involved in Ethanol‐Induced Microtia Pathogenesis Based on a Human Genome Database: GeneCards.” International Journal of Pediatric Otorhinolaryngology 142: 110595. 10.1016/j.ijporl.2020.110595. 33418206

[fsn370763-bib-0020] Loh, C. Y. , J. Y. Chai , T. F. Tang , et al. 2019. “The E‐Cadherin and N‐Cadherin Switch in Epithelial‐to‐Mesenchymal Transition: Signaling, Therapeutic Implications, and Challenges.” Cells 8, no. 10: 1118. 10.3390/cells8101118. 31547193 PMC6830116

[fsn370763-bib-0021] Martin, A. R. , and W. H. Finlay . 2015. “Nebulizers for Drug Delivery to the Lungs.” Expert Opinion on Drug Delivery 12, no. 6: 889–900. 10.1517/17425247.2015.995087. 25534396

[fsn370763-bib-0022] Mims, J. W. 2015. “Asthma: Definitions and Pathophysiology.” International Forum of Allergy & Rhinology 5, no. Suppl 1: S2–S6. 10.1002/alr.21609. 26335832

[fsn370763-bib-0023] Misiukiewicz‐Stepien, P. , and M. Paplinska‐Goryca . 2021. “Biological Effect of PM(10) on Airway Epithelium‐Focus on Obstructive Lung Diseases.” Clinical Immunology 227: 108754. 10.1016/j.clim.2021.108754. 33964432

[fsn370763-bib-0024] Mitamura, Y. , M. Murai , C. Mitoma , and M. Furue . 2018. “NRF2 Activation Inhibits Both TGF‐beta1‐ and IL‐13‐Mediated Periostin Expression in Fibroblasts: Benefit of Cinnamaldehyde for Antifibrotic Treatment.” Oxidative Medicine and Cellular Longevity 2018: 2475047. 10.1155/2018/2475047. 30186543 PMC6112270

[fsn370763-bib-0025] Pain, M. , O. Bermudez , P. Lacoste , et al. 2014. “Tissue Remodelling in Chronic Bronchial Diseases: From the Epithelial to Mesenchymal Phenotype.” European Respiratory Review 23, no. 131: 118–130. 10.1183/09059180.00004413. 24591669 PMC9487272

[fsn370763-bib-0026] Palmans, E. , R. A. Pauwels , and J. C. Kips . 2002. “Repeated Allergen Exposure Changes Collagen Composition in Airways of Sensitised Brown Norway Rats.” European Respiratory Journal 20, no. 2: 280–285. 10.1183/09031936.02.00255402. 12212956

[fsn370763-bib-0027] Park, N. , S. J. Park , M. H. Kim , and W. M. Yang . 2022. “Efficacy and Mechanism of Essential Oil From *Abies holophylla* Leaf on Airway Inflammation in Asthma: Network Pharmacology and In Vivo Study.” Phytomedicine 96: 153898. 10.1016/j.phymed.2021.153898. 35026513

[fsn370763-bib-0028] Poulalhon, N. , D. Farge , N. Roos , et al. 2006. “Modulation of Collagen and MMP‐1 Gene Expression in Fibroblasts by the Immunosuppressive Drug Rapamycin. A Direct Role as an Antifibrotic Agent?” Journal of Biological Chemistry 281, no. 44: 33045–33052. 10.1074/jbc.M606366200. 16914544

[fsn370763-bib-0029] Rubin, B. K. 2011. “Pediatric Aerosol Therapy: New Devices and New Drugs.” Respiratory Care 56, no. 9: 1411–1421; discussion 1421‐1413. 10.4187/respcare.01246. 21944688

[fsn370763-bib-0030] Safran, M. , I. Dalah , J. Alexander , et al. 2010. “GeneCards Version 3: The Human Gene Integrator.” Database: The Journal of Biological Databases and Curation 2010: baq020. 10.1093/database/baq020. 20689021 PMC2938269

[fsn370763-bib-0031] Shergis, J. L. , L. Wu , A. L. Zhang , X. Guo , C. Lu , and C. C. Xue . 2016. “Herbal Medicine for Adults With Asthma: A Systematic Review.” Journal of Asthma 53, no. 6: 650–659. 10.3109/02770903.2015.1101473. 27172294

[fsn370763-bib-0032] Sobieraj, D. M. , E. R. Weeda , E. Nguyen , et al. 2018. “Association of Inhaled Corticosteroids and Long‐Acting Beta‐Agonists as Controller and Quick Relief Therapy With Exacerbations and Symptom Control in Persistent Asthma: A Systematic Review and Meta‐Analysis.” JAMA 319, no. 14: 1485–1496. 10.1001/jama.2018.2769. 29554195 PMC5876810

[fsn370763-bib-0033] Sun, Z. , H. Wang , J. Wang , L. Zhou , and P. Yang . 2014. “Chemical Composition and Anti‐Inflammatory, Cytotoxic and Antioxidant Activities of Essential Oil From Leaves of *Mentha piperita* Grown in China.” PLoS One 9, no. 12: e114767. 10.1371/journal.pone.0114767. 25493616 PMC4262447

[fsn370763-bib-0034] Takizawa, H. 2015. “Impacts of Particulate Air Pollution on Asthma: Current Understanding and Future Perspectives.” Recent Patents on Inflammation & Allergy Drug Discovery 9, no. 2: 128–135. 10.2174/1872213x09666150623110714. 26100558

[fsn370763-bib-0035] Tao, Q. , J. Du , X. Li , et al. 2020. “Network Pharmacology and Molecular Docking Analysis on Molecular Targets and Mechanisms of Huashi Baidu Formula in the Treatment of COVID‐19.” Drug Development and Industrial Pharmacy 46, no. 8: 1345–1353. 10.1080/03639045.2020.1788070. 32643448 PMC7441778

[fsn370763-bib-0036] Wang, J. , Y. Zhou , H. Zhang , et al. 2023. “Pathogenesis of Allergic Diseases and Implications for Therapeutic Interventions.” Signal Transduction and Targeted Therapy 8, no. 1: 138. 10.1038/s41392-023-01344-4. 36964157 PMC10039055

[fsn370763-bib-0037] Wang, W. , Q. Yao , F. Teng , J. Cui , J. Dong , and Y. Wei . 2021. “Active Ingredients From Chinese Medicine Plants as Therapeutic Strategies for Asthma: Overview and Challenges.” Biomedicine & Pharmacotherapy 137: 111383. 10.1016/j.biopha.2021.111383. 33761604

[fsn370763-bib-0038] Wu, H. , J. Li , F. Zhang , L. Li , Z. Liu , and Z. He . 2012. “Essential Oil Components From *Asarum sieboldii* Miquel Are Toxic to the House Dust Mite *Dermatophagoides farinae* .” Parasitology Research 111, no. 5: 1895–1899. 10.1007/s00436-012-3032-5. 22833176

[fsn370763-bib-0039] Wu, J. , Y. Liu , J. Hu , J. Xie , Z. Nie , and W. Yin . 2020. “Protective Activity of Asatone Against Ovalbumin‐Induced Allergic Asthma.” International Journal of Clinical and Experimental Pathology 13, no. 10: 2487–2494. 33165354 PMC7642709

[fsn370763-bib-0040] Yamauchi, K. , and H. Inoue . 2007. “Airway Remodeling in Asthma and Irreversible Airflow Limitation‐ECM Deposition in Airway and Possible Therapy for Remodeling.” Allergology International 56, no. 4: 321–329. 10.2332/allergolint.R-07-151. 17965575

[fsn370763-bib-0041] Yang, J. , B. Song , and J. Wu . 2022. “Herbal Nanoformulations for Asthma Treatment.” Current Pharmaceutical Design 28, no. 1: 46–57. 10.2174/1381612827666210929113528. 34587880

[fsn370763-bib-0042] Yang, L. , X. Y. Hou , Y. Wei , P. Thai , and F. Chai . 2017. “Biomarkers of the Health Outcomes Associated With Ambient Particulate Matter Exposure.” Science of the Total Environment 579: 1446–1459. 10.1016/j.scitotenv.2016.11.146. 27908628

[fsn370763-bib-0043] Zdanowicz, M. M. 2007. “Pharmacotherapy of Asthma.” American Journal of Pharmaceutical Education 71, no. 5: 98. 10.5688/aj710598. 17998995 PMC2064896

[fsn370763-bib-0044] Zhang, X. , X. Li , P. Wang , S. Zhao , and Y. Zhao . 2025. “Safranal Restores RUNX3‐Mediated Immunoregulation by Inhibiting the NLRP3 Inflammasome in Allergic Asthma.” Naunyn‐Schmiedeberg's Archives of Pharmacology. 10.1007/s00210-025-03943-0. 40163148

