# Supplementary figures and images for: Effectiveness of Inhalation of a New Essential Oil Formulation on Asthma Through Network Pharmacology and In Vivo Analysis
Source: Food Sci Nutr. 2025 Aug 7;13(8):e70763. doi: 10.1002/fsn3.70763 (PMC12329566; doi:10.1002/fsn3.70763)

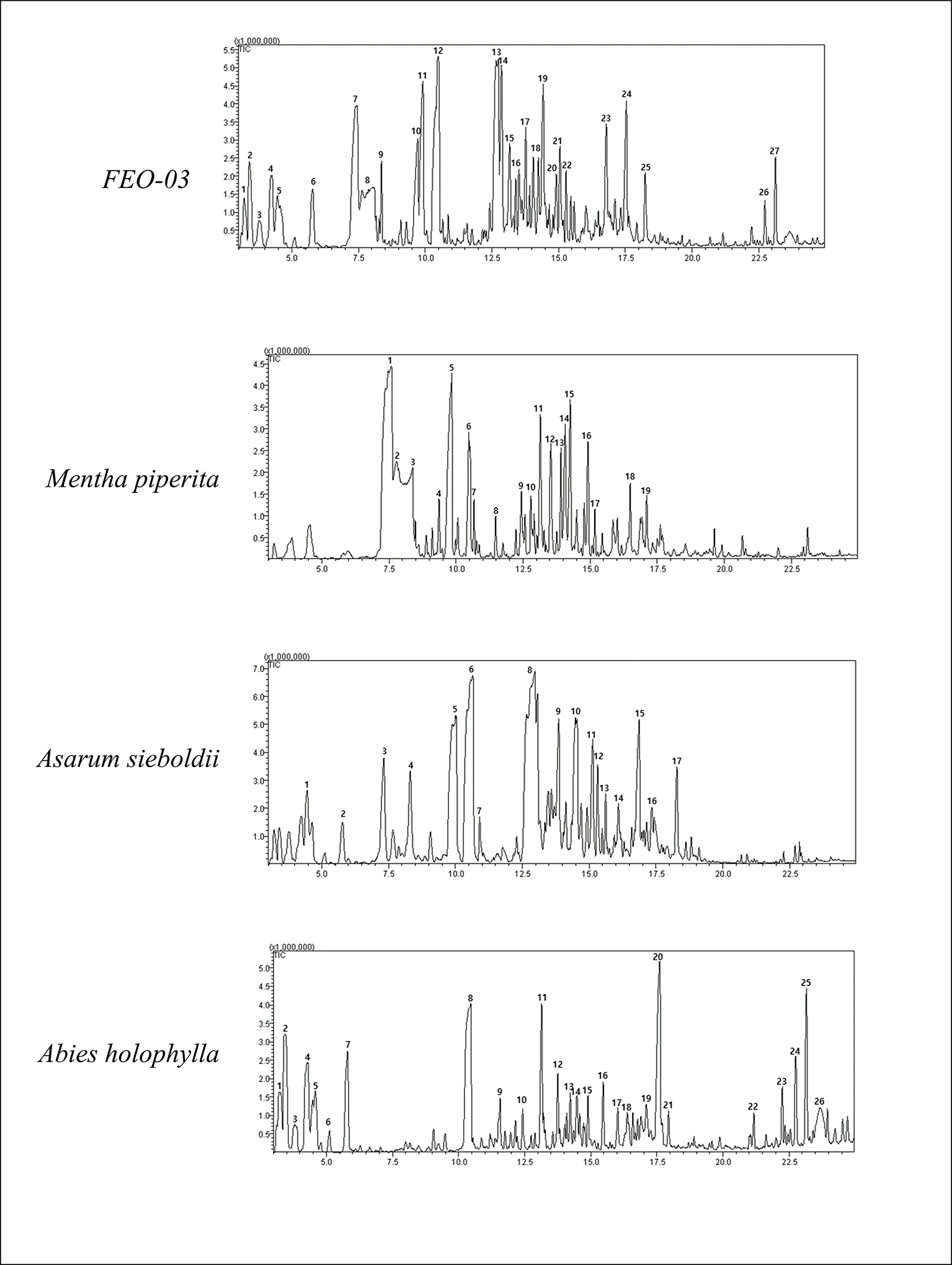

Supplement: Supplementary file 1 — Figure S1: fsn370763‐sup‐0001‐FigureS1.tif. [file FSN3-13-e70763-s001.tif]
