# Supplementary material for: Effectiveness of Inhalation of a New Essential Oil Formulation on Asthma Through Network Pharmacology and In Vivo Analysis
Source: Food Sci Nutr. 2025 Aug 7;13(8):e70763. doi: 10.1002/fsn3.70763 (PMC12329566; doi:10.1002/fsn3.70763)
Supplement: Supplementary file 2 — Table S1: fsn370763‐sup‐0002‐TablesS1‐S10.zip. Table S2: fsn370763‐sup‐0002‐TablesS1‐S10.zip. Table S3: fsn370763‐sup‐0002‐TablesS1‐S10.zip. Table S4: fsn370763‐sup‐0002‐TablesS1‐S10.zip. Table S5: fsn370763‐sup‐0002‐TablesS1‐S10.zip. Table S6: fsn370763‐sup‐0002‐TablesS1‐S10.zip. Table S7: fsn370763‐sup‐0002‐TablesS1‐S10.zip. Table S8: fsn370763‐sup‐0002‐TablesS1‐S10.zip. Table S9: fsn370763‐sup‐0002‐TablesS1‐S10.zip. Table S10: fsn370763‐sup‐0002‐TablesS1‐S10.zip. [file FSN3-13-e70763-s002.zip › fsn370763-sup-0003-TableS9-10.docx]

**Supplementary table 9. Primer sequences for RT-PCR**

| **Species** | **Genes** | **Forward primers (5’-3’)** | **Reverse primers (5’-3’)** |
| --- | --- | --- | --- |
| Mouse | *Il13* | ACCACGGTCATTGCTCTCA | GTGTCTCGGACATGCAAGCT |
|  | *Il4* | ATGGGTCTCAACCCCCAGC | GCTCTTTACGCTTTCCAGGAAGTC |
|  | *Il5* | ATGATCGTGCCTCTGTGCCTGGAGC | CTGTTTTTCCTGGAGTAAACTGGGG |
|  | *Tnfa* | TACTGAACTTCGGGGTGATTGGTCC | CAGCCTTGTCCCTTGAAGAGAACC |
|  | *Il1b* | CAGGATGAGGACATGAGCACC | CTCTGCACACTCAAACTCCAC |
|  | *Il6* | CGGAGAGGAGACTTCACAGAGGA | GGAGAGCATTGGAAATTGGGG |
|  | *Col1a1* | ATGGGTCTCAACCCCCAGC | GCTCTTTACGCTTTCCAGGAAGTC |
|  | *Col1a3* | ACGTAAGCACTGGTGGACAG | CAGGAGGGCCATAGCTGAAC |
|  | *Postn* | TAGCCCAATTAGGCTTGGCATCC | TAAGAAGGCGTTGGTCCATGCT |
|  | *Tgfb* | CGGGGCGACCTGGGCACCATCCATGAC | CTGCTCCACCTTGGGCTTGCGACCCAC |
|  | *Gapdh* | GGCATGGACTGTGGTCATGA | TTCACCACCATGGAGAAGGC |

**Supplementary table 10. List of antibodies used in western blotting**

| **Primary antibody(dillution)** | **Source** | **Catalogue** | **Manufacture** |
| --- | --- | --- | --- |
| N-cadherin (1:1000) | Rabbit | # 4061 | Cell signaling |
| E-cadherin (1:1000) | Mouse | # 14472 | Cell signaling |
| β-actin (1:1000) | Rabbit | # 4967 | Cell signaling |
